# Supplementary material for: Design, Synthesis, and Biological Evaluation of Novel Dihydropyridine and Pyridine Analogs as Potent Human Tissue Nonspecific Alkaline Phosphatase Inhibitors with Anticancer Activity: ROS and DNA Damage-Induced Apoptosis
Source: Molecules. 2022 Sep 22;27(19):6235. doi: 10.3390/molecules27196235 (PMC9570995; doi:10.3390/molecules27196235)
Supplement: Supplementary file 1 [file molecules-27-06235-s001.zip › molecules-1850593-supplementary.pdf]

Supporting Information

Design, Synthesis and Biological Evaluation of Novel Dihydropyridine and Pyridine Analogs as Potent Human Tissue Nonspecific Alkaline Phosphatase Inhibitors with Anticancer Activity: ROS and DNA Damage-Induced Apoptosis

Nazeer Ahmad Khan[1], Faisal Rashid[2], Muhammad Siraj Khan Jadoon[2], Saquib Jalil[2], Zulfiqar Ali Khan[3], Raha Orfali[4], Shagufta Perveen[5], Areej Al-Taweel[4], Jamshed Iqbal[2] and Sohail Anjum Shahzad[1,*]

[1]Department of Chemistry, COMSATS University Islamabad, Abbottabad Campus, University Road, Abbottabad 22060, Pakistan

[2]Centre for Advanced Drug Research, COMSATS University Islamabad, Abbottabad Campus, Abbot-tabad 22060, Pakistan

[3]Department of Chemistry, Government College University, Faisalabad 38000, Pakistan

[4]Department of Pharmacognosy, College of Pharmacy, King Saud University, P.O. Box 2457, Riyadh 11451, Saudi Arabia

[5]Department of Chemistry, School of Computer, Mathematical and Natural Sciences, Morgan State University, Baltimore, MD 21251, USA

*Department of Chemistry, COMSATS University Islamabad, Abbottabad Campus, University Road, Abbottabad 22060, Pakistan (Sohail Anjum Shahzad). E-mail: sashahzad@cuiatd.edu.pk; sohail_chem@yahoo.com (Sohail Anjum Shahzad).

HPLC Analysis Report

SHIMADZU LabSolutions

<Sample Information>

Sample Name	: NAk 45-6		
Sample ID	: NAk 45-6		
Data Filename	: NAk 45-6 Data1.lcd		
Method Filename	: NAk 43-2.lcm		
Batch Filename	:		
Vial #	: 1-1	Sample Type	: Unknown
Injection Volume	: 20 uL		
Date Acquired	: 01-Sep-22 3:48:46 PM	Acquired by	: System Administrator
Date Processed	: 01-Sep-22 4:13:52 PM	Processed by	: System Administrator

<Chromatogram>

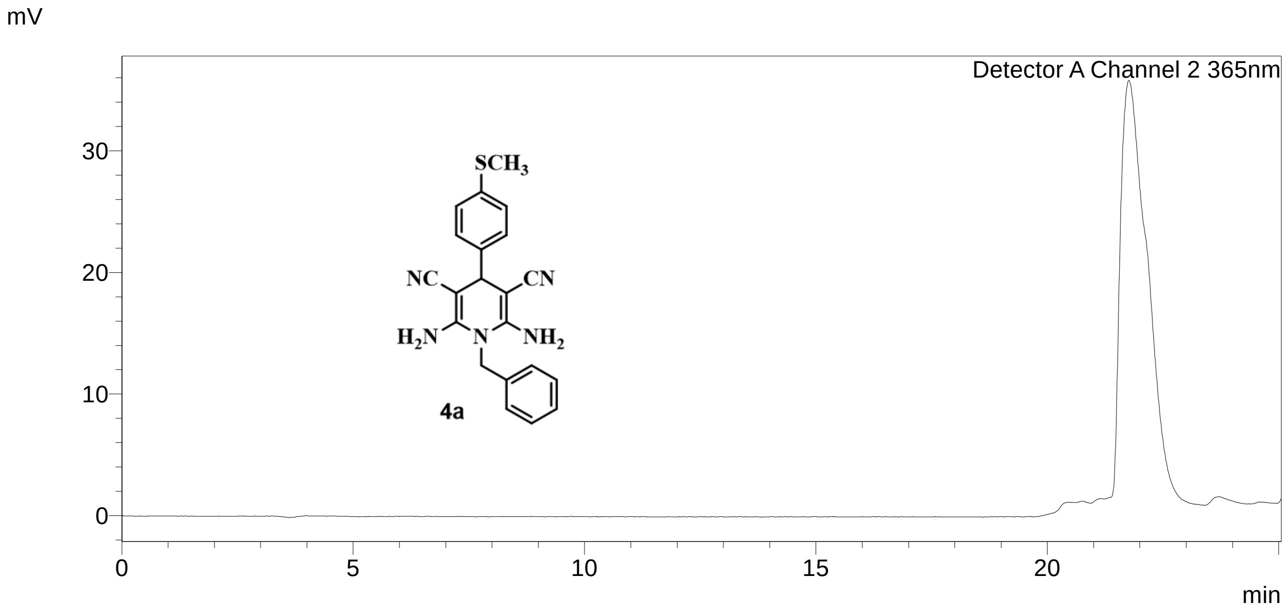

Detector A Channel 2 365nm

Peak#	Ret. Time	Area	Height	Conc.	Unit	Mark	Name
1	3.989	2019	147	0.000			
2	4.260	1221	122	0.000		V	
3	4.495	1676	114	0.000		V	
4	4.835	1070	92	0.000		V	
5	20.452	18886	1047	0.000			
6	20.533	4547	1020	0.000		V	
7	20.755	21756	1076	0.000		V	
8	21.159	19113	1193	0.000		V	
9	21.761	1482871	35436	0.000		SV	
10	23.714	17902	670	0.000		T	
11	24.581	1165	150	0.000		TV	
12	24.650	1223	139	0.000		TV	
Total		1573449	41207				

SHIMADZU LabSolutions

Analysis Report

<Sample Information>

Sample Name	: NAK 48-29		
Sample ID	: NAK 48-29		
Data Filename	: NAK 48-29 Data1.lcd		
Method Filename	: NAK 48-20.lcm		
Batch Filename	:		
Vial #	: 1-1	Sample Type	: Unknown
Injection Volume	: 20 uL		
Date Acquired	: 03-Sep-22 7:35:09 PM	Acquired by	: System Administrator
Date Processed	: 03-Sep-22 8:10:19 PM	Processed by	: System Administrator

<Chromatogram>

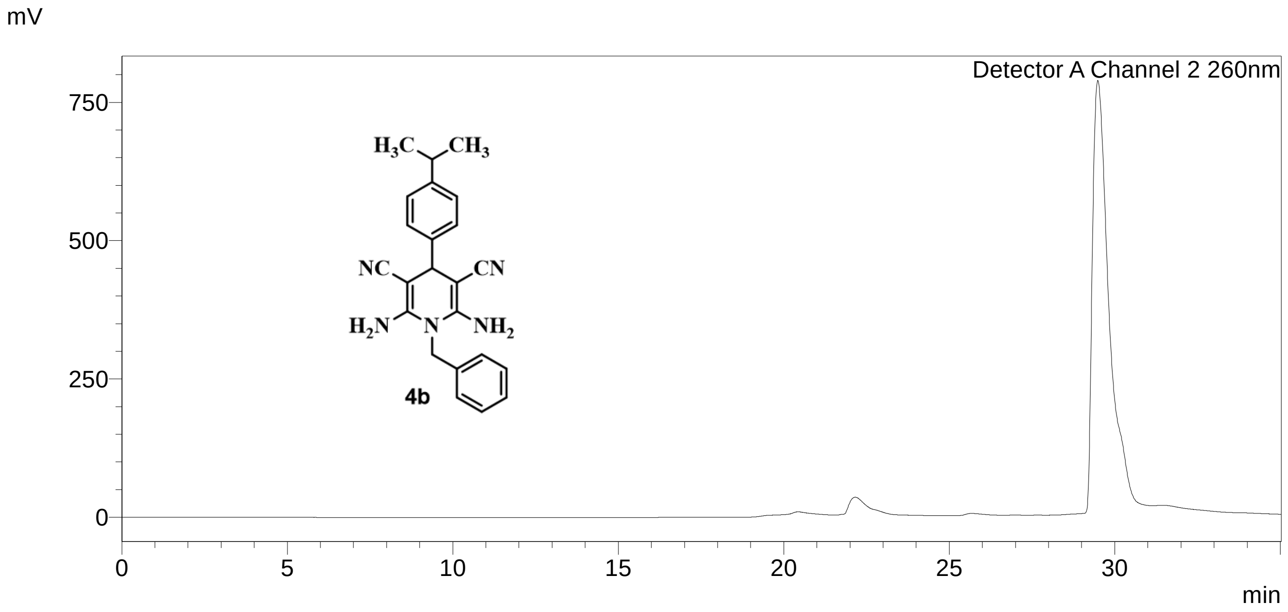

Detector A Channel 2 260nm

Peak#	Ret. Time	Area	Height	Conc.	Unit	Mark	Name
1	3.460	8031	308	0.000		V	
2	3.942	1300	91	0.000		V	
3	20.432	700312	9319	0.000			
4	22.158	1752917	35176	0.000		SV	
5	25.671	209942	4602	0.000		V	
6	26.971	46559	1227	0.000		V	
7	27.670	24679	870	0.000		V	
8	29.487	30089281	785967	0.000		SV	
9	31.470	105736	2576	0.000		T	
Total		32938755	840135				

SHIMADZU LabSolutions

Analysis Report

<Sample Information>

Sample Name	: NAk 46-8		
Sample ID	: NAk 46-8		
Data Filename	: NAk 46-8 Data1.lcd		
Method Filename	: NAk 43-2.lcm		
Batch Filename	:		
Vial #	: 1-1	Sample Type	: Unknown
Injection Volume	: 20 uL		
Date Acquired	: 01-Sep-22 5:11:02 PM	Acquired by	: System Administrator
Date Processed	: 01-Sep-22 5:17:24 PM	Processed by	: System Administrator

<Chromatogram>

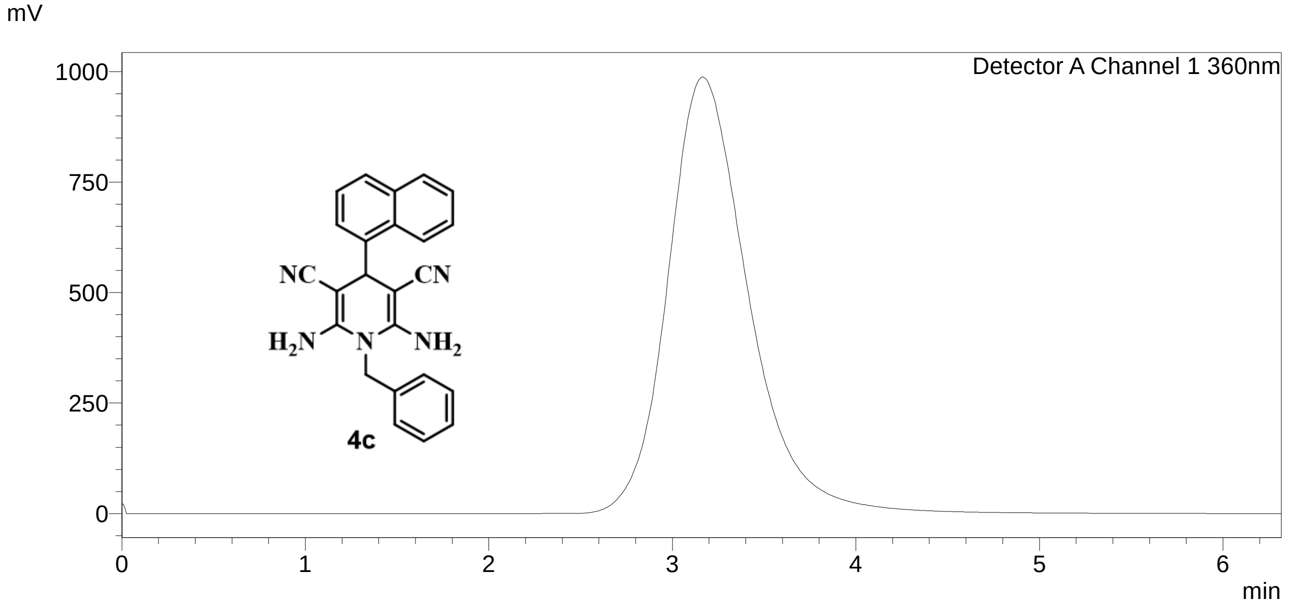

<Peak Table>

Detector A Channel 1 360nm

Peak#	Ret. Time	Area	Height	Conc.	Unit	Mark	Name
1	3.165	30188947	988256	0.000		V	
Total		30188947	988256				

SHIMADZU LabSolutions

Analysis Report

<Sample Information>

Sample Name	: NAk 47-16		
Sample ID	: NAk 47-16		
Data Filename	: NAk 47-16 Data1.lcd		
Method Filename	: NAk 43-2.lcm		
Batch Filename	:		
Vial #	: 1-1	Sample Type	: Unknown
Injection Volume	: 10 uL		
Date Acquired	: 01-Sep-22 7:03:42 PM	Acquired by	: System Administrator
Date Processed	: 01-Sep-22 7:21:49 PM	Processed by	: System Administrator

<Chromatogram>

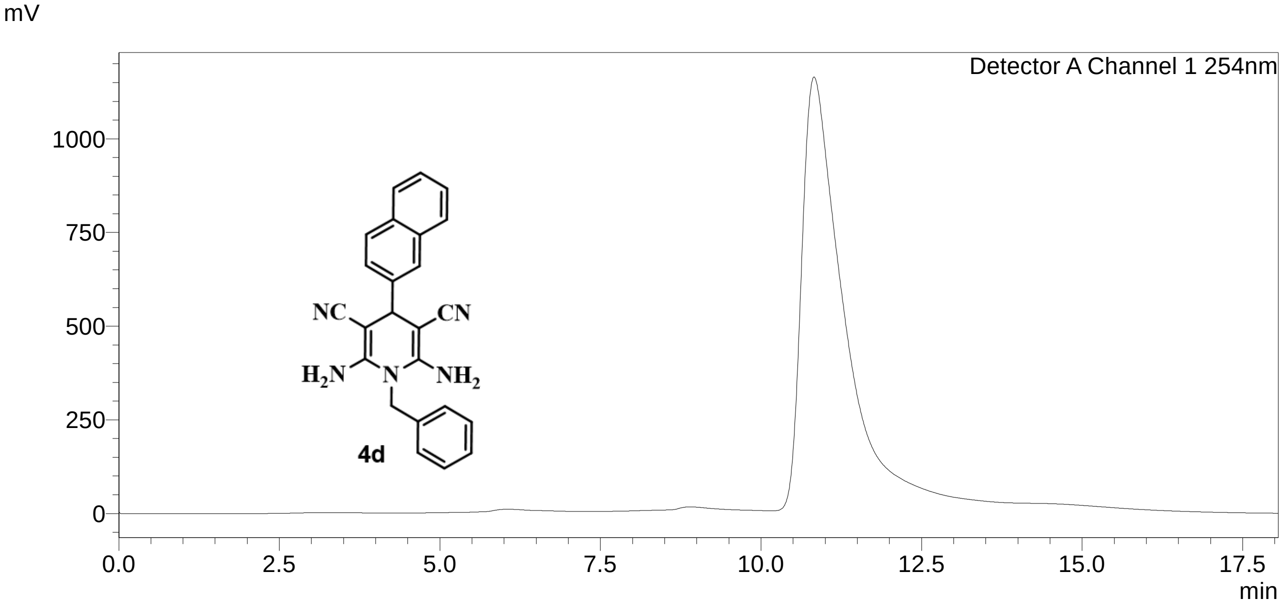

<Peak Table>

Detector A Channel 1 254nm

Peak#	Ret. Time	Area	Height	Conc.	Unit	Mark	Name
1	3.233	198014	2364	0.000		V	
2	6.058	938543	11384	0.000		V	
3	8.897	1626638	17427	0.000		V	
4	10.826	59582250	1164738	0.000		V	
Total		62345445	1195914				

SHIMADZU LabSolutions

Analysis Report

<Sample Information>

Sample Name	: NAK 58-25		
Sample ID	: NAK 58-25		
Data Filename	: NAK 58-25 Data1.lcd		
Method Filename	: NAK 48-20.lcm		
Batch Filename	:		
Vial #	: 1-1	Sample Type	: Unknown
Injection Volume	: 20 uL		
Date Acquired	: 02-Sep-22 9:27:08 PM	Acquired by	: System Administrator
Date Processed	: 02-Sep-22 10:07:18 PM	Processed by	: System Administrator

<Chromatogram>

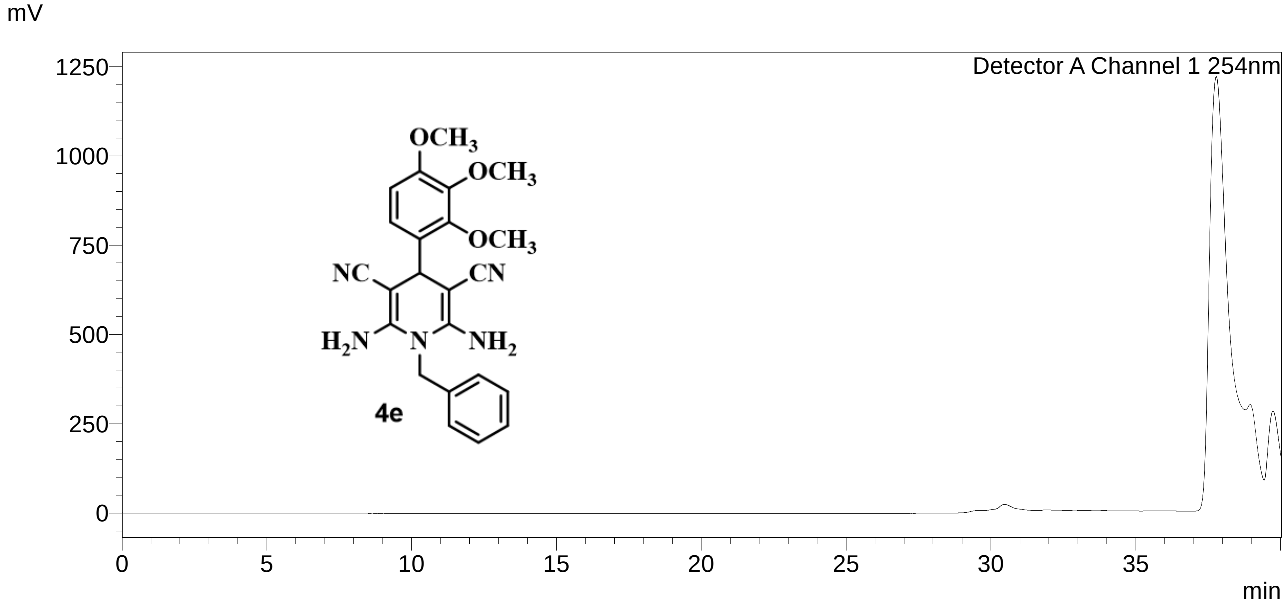

<Peak Table>

Detector A Channel 1 254nm

Peak#	Ret. Time	Area	Height	Conc.	Unit	Mark	Name
1	3.341	14369	570	0.000			
2	30.466	1398394	22785	0.000			
3	31.920	146666	5755	0.000		V	
4	32.192	169363	4891	0.000		V	
5	33.170	58714	2518	0.000		V	
6	33.605	123002	3268	0.000		V	
7	35.534	10668	412	0.000			
8	36.123	14248	442	0.000		V	
9	37.778	52149086	1191346	0.000			
10	38.954	5548546	229285	0.000		V	
11	39.737	2897453	151755	0.000			
Total		62530509	1613028				

[illegible]

SHIMADZU LabSolutions

Analysis Report

<Sample Information>

Sample Name	: NAk 43-4		
Sample ID	: NAk 43-4		
Data Filename	: NAk 43-4 Data1.lcd		
Method Filename	: NAk 43-2.lcm		
Batch Filename	:		
Vial #	: 1-1	Sample Type	: Unknown
Injection Volume	: 20 uL		
Date Acquired	: 01-Sep-22 1:52:18 PM	Acquired by	: System Administrator
Date Processed	: 01-Sep-22 2:17:25 PM	Processed by	: System Administrator

<Chromatogram>

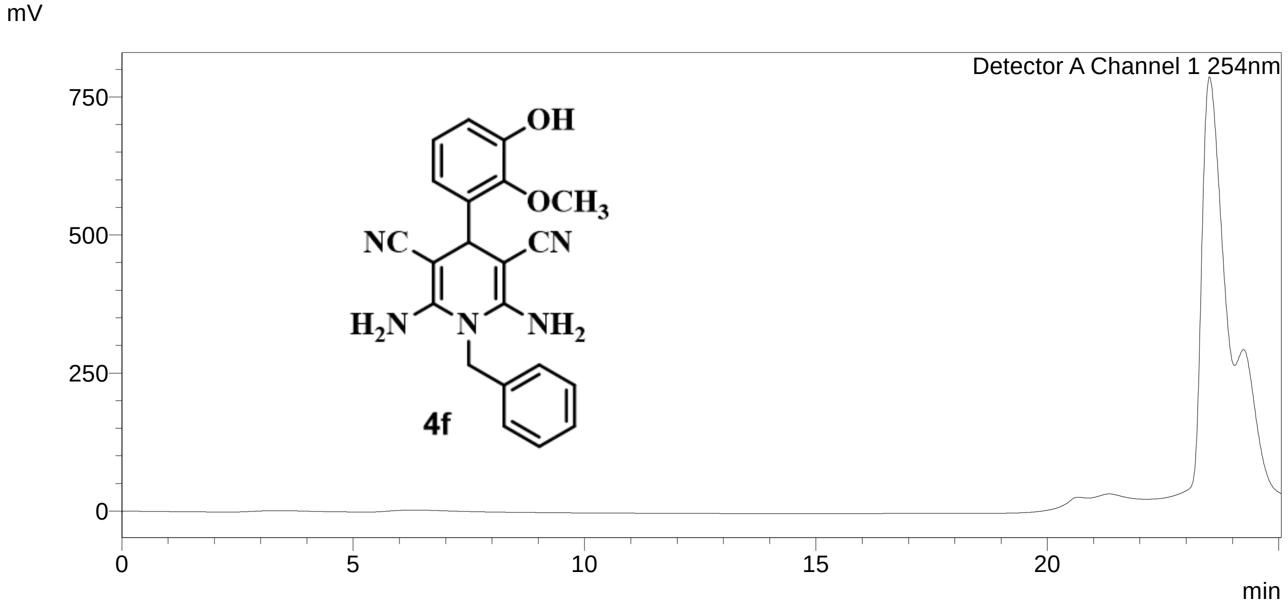

<Peak Table>

Detector A Channel 1 254nm

Peak#	Ret. Time	Area	Height	Conc.	Unit	Mark	Name
1	3.359	215396	2519	0.000			
2	6.321	450041	4171	0.000			
3	17.117	2745	96	0.000		V	
4	17.292	1241	95	0.000		V	
5	17.567	1907	85	0.000		V	
6	20.668	632673	20225	0.000			
7	21.338	1174748	22034	0.000		V	
8	23.503	24863802	762873	0.000		V	
9	24.239	7724167	264250	0.000		V	
Total		35066721	1076348				

SHIMADZU LabSolutions

Analysis Report

<Sample Information>

Sample Name	: NAK 49-21		
Sample ID	: NAK 49-21		
Data Filename	: NAK 49-21 Data1.lcd		
Method Filename	: NAK 48-20.lcm		
Batch Filename	:		
Vial #	: 1-1	Sample Type	: Unknown
Injection Volume	: 20 uL		
Date Acquired	: 02-Sep-22 5:25:43 PM	Acquired by	: System Administrator
Date Processed	: 02-Sep-22 6:05:53 PM	Processed by	: System Administrator

<Chromatogram>

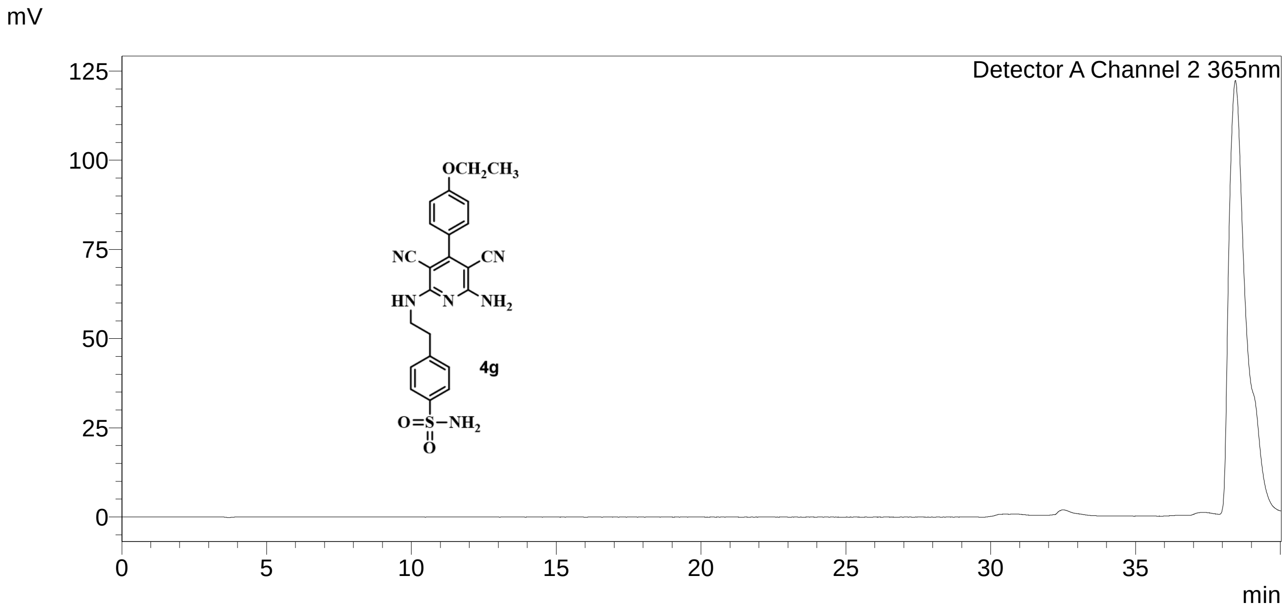

Detector A Channel 2 365nm

Peak#	Ret. Time	Area	Height	Conc.	Unit	Mark	Name
1	3.368	3574	153	0.000		V	
2	29.202	1116	83	0.000		V	
3	30.425	18348	819	0.000			
4	30.501	8449	821	0.000		V	
5	30.857	32316	840	0.000		V	
6	31.492	2054	385	0.000		V	
7	31.592	1399	354	0.000		V	
8	31.691	2368	343	0.000		V	
9	31.933	4725	370	0.000		V	
10	32.508	77098	1804	0.000		V	
11	36.408	1533	102	0.000			
12	37.233	10107	675	0.000			
13	37.341	14396	666	0.000		V	
14	38.449	5018218	121378	0.000			
Total		5195701	128794				

SHIMADZU LabSolutions

Analysis Report

<Sample Information>

Sample Name	: NAK 52-23		
Sample ID	: NAK 52-23		
Data Filename	: NAK 52-23 Data1.lcd		
Method Filename	: NAK 48-20.lcm		
Batch Filename	:		
Vial #	: 1-1	Sample Type	: Unknown
Injection Volume	: 20 uL		
Date Acquired	: 02-Sep-22 7:07:40 PM	Acquired by	: System Administrator
Date Processed	: 02-Sep-22 7:47:51 PM	Processed by	: System Administrator

<Chromatogram>

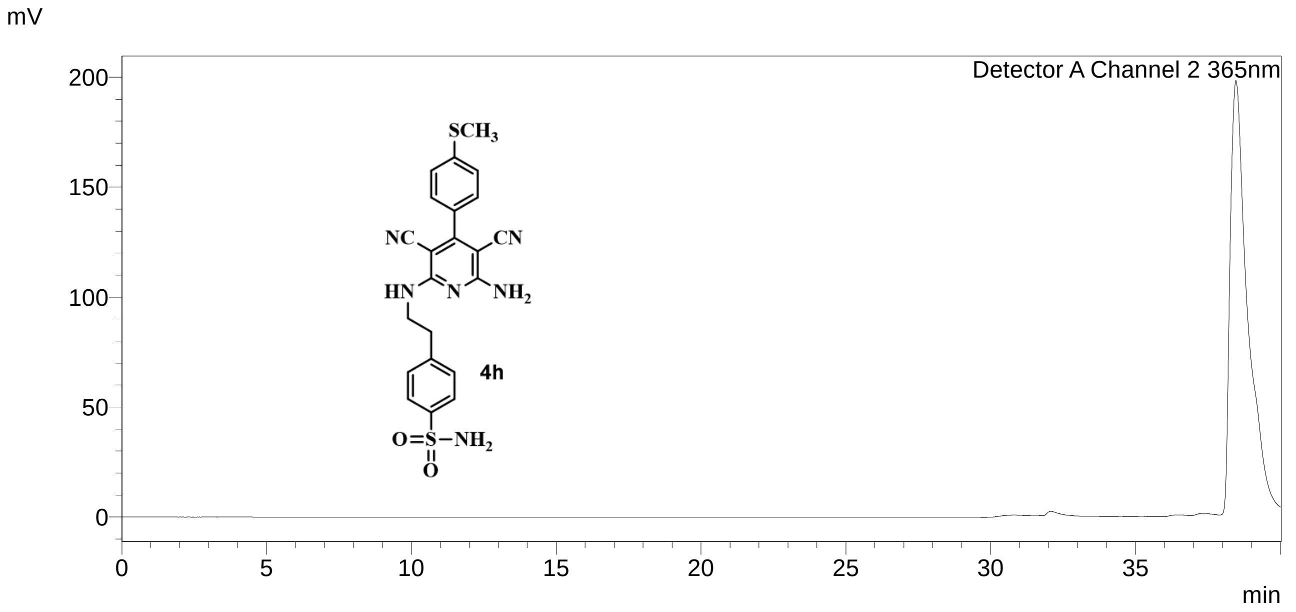

Detector A Channel 2 365nm

Peak#	Ret. Time	Area	Height	Conc.	Unit	Mark	Name
1	30.775	49070	975	0.000		V	
2	31.445	11236	739	0.000		V	
3	31.550	10551	713	0.000		V	
4	32.070	84046	2390	0.000		V	
5	36.455	11296	606	0.000			
6	36.542	8227	569	0.000		V	
7	37.348	32613	970	0.000		V	
8	38.470	8178111	196627	0.000			
Total		8385151	203589				

SHIMADZU LabSolutions

Analysis Report

<Sample Information>

Sample Name	: NAK 50-22		
Sample ID	: NAK 50-22		
Data Filename	: NAK 50-22 Data1.lcd		
Method Filename	: NAK 48-20.lcm		
Batch Filename	:		
Vial #	: 1-1	Sample Type	: Unknown
Injection Volume	: 20 uL		
Date Acquired	: 02-Sep-22 6:16:35 PM	Acquired by	: System Administrator
Date Processed	: 02-Sep-22 6:56:45 PM	Processed by	: System Administrator

<Chromatogram>

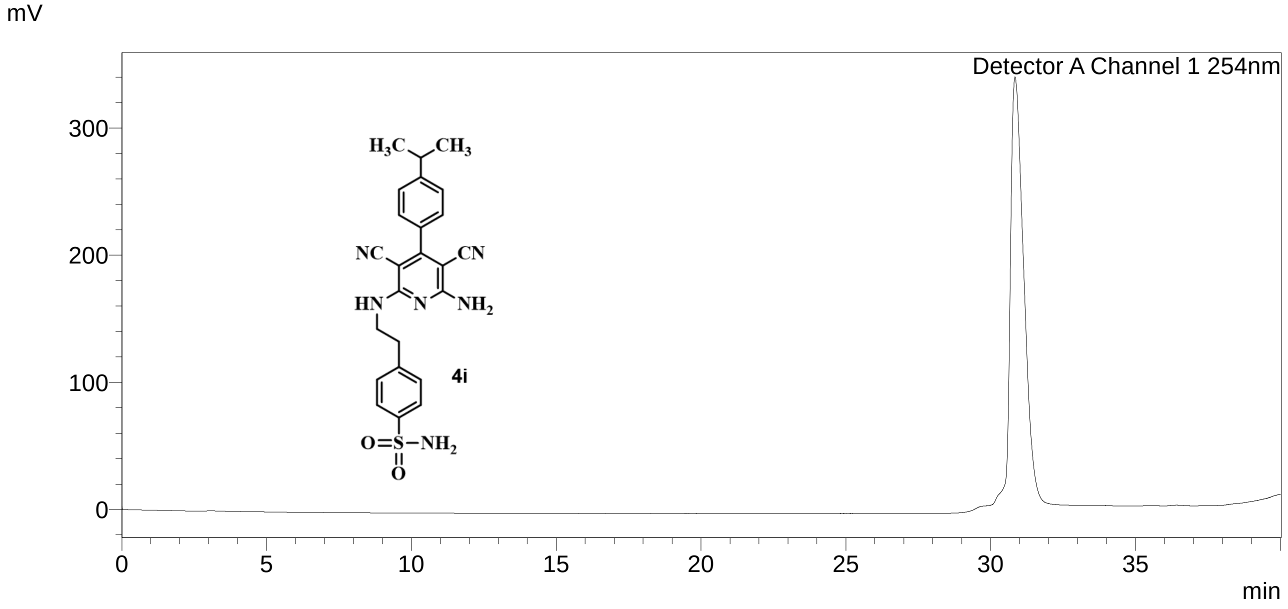

<Peak Table>

Detector A Channel 1 254nm

Peak#	Ret. Time	Area	Height	Conc.	Unit	Mark	Name
1	3.052	7908	213	0.000			
2	30.841	11865219	340893	0.000		S	
3	33.703	9742	315	0.000		TV	
4	35.523	8641	259	0.000			
5	36.431	18340	607	0.000		V	
6	37.467	1438	53	0.000			
Total		11911288	342340				

SHIMADZU LabSolutions Analysis Report

<Sample Information>

Sample Name	: NAK 55-24
Sample ID	: NAK 55-24
Data Filename	: NAK 55-24 Data1.lcd
Method Filename	: NAK 48-20.lcm
Batch Filename	:
Vial #	: 1-1
Sample Type	: Unknown
Injection Volume	: 20 uL
Date Acquired	: 02-Sep-22 8:29:29 PM
Acquired by	: System Administrator
Date Processed	: 02-Sep-22 9:09:40 PM
Processed by	: System Administrator

<Chromatogram>

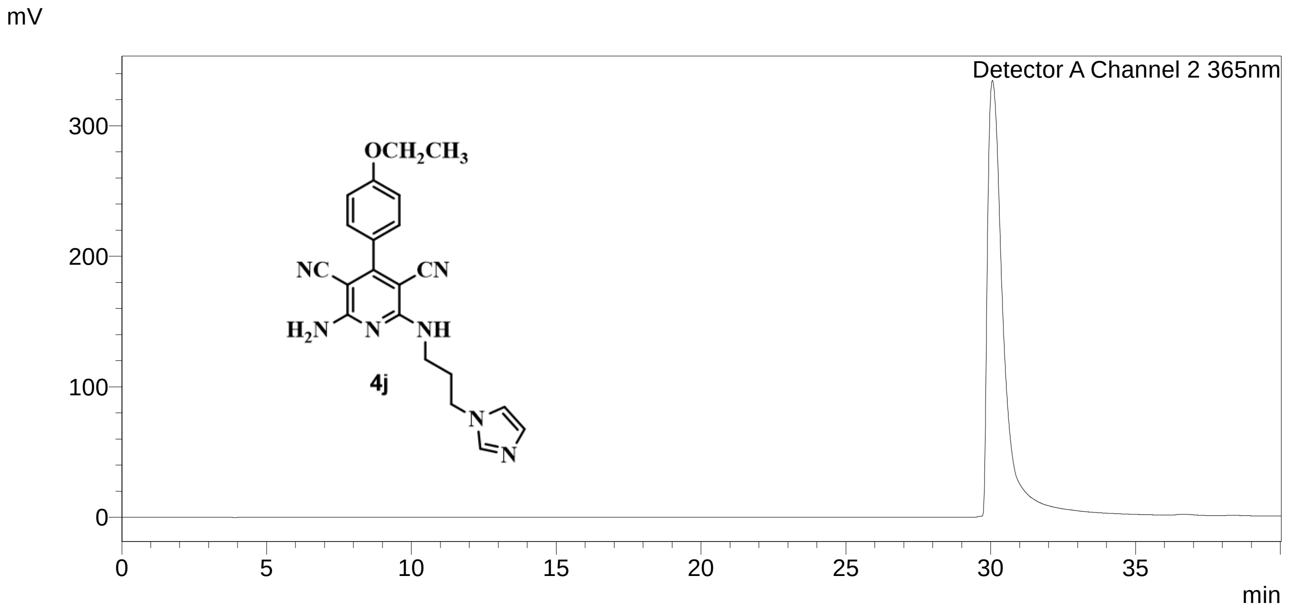

Detector A Channel 2 365nm

Peak#	Ret. Time	Area	Height	Conc.	Unit	Mark	Name
1	3.358	2092	141	0.000		V	
2	3.630	2061	190	0.000		V	
3	4.158	1193	123	0.000			
4	30.060	13563798	334741	0.000		S	
5	36.258	1775	162	0.000		TV	
6	36.625	29734	803	0.000		TV	
7	38.367	16171	474	0.000		T	
8	39.125	1075	88	0.000		TV	
Total		13617900	336721				
